# The Effect of the Combination of Temozolomide and Flubendazole on Glioblastoma Cells

**DOI:** 10.3390/cells15141239

**Published:** 2026-07-09

**Authors:** Katerina Kapickova, Barbora Vitovcova, Veronika Skarkova, Radim Havelek, Adam Skarka, Hana Vosmikova, Jiri Soukup, Eliska Kohoutova, Petra Matouskova, Emil Rudolf

**Affiliations:** 1 Department of Medical Biology and Genetics, Faculty of Medicine in Hradec Kralove, Charles University, Simkova 870, 500 03 Hradec Kralove, Czech Republicrudolf@lfhk.cuni.cz (E.R.); 2 Department of Biology, Faculty of Medicine in Pilsen, Charles University, Alej Svobody 1655/76, 323 00 Pilsen, Czech Republic; 3 Department of Medical Biochemistry, Faculty of Medicine in Hradec Kralove, Charles University, Simkova 870, 500 03 Hradec Kralove, Czech Republic; 4 Department of Chemistry, Faculty of Sciences, University of Hradec Kralove, Hradecka 1285, 500 03 Hradec Kralove, Czech Republic; 5 The Fingerland Department of Pathology, University Hospital Hradec Kralove, Sokolska 581, 500 05 Hradec Kralove, Czech Republic; 6 Department of Pathology, Military University Hospital Prague, U Vojenske Nemocnice 1200, 169 02 Prague, Czech Republic; 7 Department of Pathology, First Faculty of Medicine and General University Hospital in Prague, Charles University, Studnickova 2039, 128 00 Prague, Czech Republic; 8 Department of Biochemical Sciences, Faculty of Pharmacy, Charles University, Akademika Heyrovskeho 1203, 500 03 Hradec Kralove, Czech Republicmatousp7@faf.cuni.cz (P.M.)

**Keywords:** glioblastoma, temozolomide, flubendazole, microtubules, cell cycle, combination therapy

## Abstract

**Highlights:**

**What are the main findings?**

- The combination of temozolomide and flubendazole reduced glioblastoma cell proliferation compared with the untreated control group and TMZ monotherapy.
- FLU-containing treatments were associated with altered microtubule organization, caspase activation, G2/M cell cycle accumulation, and changes in drug accumulation in vitro and in vivo.

**What are the implications of the main findings?**

- Microtubule targeting may modulate the cellular response of glioblastoma cells to temozolomide.
- Temozolomide and flubendazole co-treatment should be further validated to clarify its pharmacological interaction and potential relevance for glioblastoma therapy.

**Abstract:**

Glioblastoma multiforme (GBM) is a highly aggressive brain tumor characterized by rapid development of chemoresistance and poor patient survival. Due to the limited efficacy of current therapeutic protocols, alternative approaches are needed. One potential strategy is combined treatment with DNA-damaging and microtubule-targeting agents. This study investigated the biological effects of temozolomide (TMZ) and flubendazole (FLU) co-treatment in cultured GBM cells. Three GBM cell lines (A172, T98G, and U118MG) with different molecular profiles were treated with TMZ, FLU, or their combination. Treatment effects were evaluated using WST-1 assay, microscopy, flow cytometry, RT-PCR, and Western blotting. Drug accumulation in tumor and brain tissues was additionally analyzed in a nu-nu mouse model using LC/MS. Combined TMZ and FLU treatment reduced cell proliferation compared with untreated control and TMZ monotherapy in the tested GBM cell lines. FLU-containing treatments were associated with morphological alterations, caspase activation, altered microtubule organization, and G2/M cell cycle accumulation. In addition, co-administration of TMZ and FLU affected drug accumulation in vitro and in brain and tumor tissues in vivo. These findings suggest that the microtubule targeting may modulate the response of GBM cells to TMZ. However, the pharmacological interaction and potential relevance of TMZ + FLU co-treatment require further preclinical validation.

## 1. Introduction

Glioblastoma multiforme (GBM) is a highly aggressive primary brain tumor with a very poor prognosis. Despite the treatment, including the maximal possible safe surgical resection combined with radiotherapy and chemotherapy with the alkylating drug temozolomide (TMZ), the median overall survival is only 12 to 15 months after diagnosis [1]. Infiltrative growth, high invasiveness, and many different genetic and epigenetic variants of glioblastoma cells represent chief reasons for treatment failure [2]. These factors combine with limited entry of chemotherapeutics across the blood–brain barrier (as for instance only 20% of the plasma concentration of TMZ is found in the brain tumor), TMZ-related chemoresistance and its various side effects [3].

The very poor prognosis and limited treatment effectiveness of this malignant tumor imply the critical need for new or improved treatment options [2]. In recent years, GBM treatment has increasingly relied on combination strategies that aim to overcome the limitations of monotherapies and address the complex tumor microenvironment, intratumoral heterogeneity, and blood–brain barrier-related delivery limitations. Conventional adjuvant agents such as nitrosoureas (e.g., lomustine, carmustine) have been used particularly in temozolomide-resistant cases [4], while bevacizumab, an anti-VEGF monoclonal antibody, has been employed to target tumor angiogenesis [5]. Drug repurposing approaches have introduced agents like chlorpromazine, cannabinoids, chloroquine, and tadalafil, as well as novel inhibitors such as bromodomain and extra-terminal motif inhibitors (BET), aiming to target GBM cell signaling, inhibit autophagy, and reduce inflammation [6]. Immunotherapy remains a highly investigated field despite limited efficacy in monotherapy. Checkpoint inhibitors targeting PD-1 (e.g., nivolumab) and CTLA-4 (e.g., ipilimumab) are being evaluated in combination with radiotherapy, temozolomide, or other immunostimulatory agents [7,8,9]. Other promising approaches include cancer vaccines (e.g., DCvax, EO2401, UCPVax), CAR-T cell therapies or oncolytic virus therapy (e.g., HSV-2-derived oncolytic virus), as well as the use of natural compounds, including phytotherapy and mycotherapy [6,10,11]. These efforts reflect the growing interest in multimodal strategies that target various aspects of GBM biology and the tumor immune microenvironment. The main goal is to improve therapeutic efficacy and patient survival.

Considering that the traditional new drug development processes are costly and time-intensive, the use of alternative strategies including the combination of drugs with different mechanisms of action is necessary. The administration of suitably selected drug combinations can improve the therapeutic effect, prevent or delay the development of drug resistance, and decrease the current drug dosage, leading to the reduction of side effects [12].

One of the established concepts in cancer therapy is targeting the cellular microtubules due to their key role in cell division, migration, invasion, and metastasis of cancer cells [13]. Malignant cells, in general, often display alterations in the expression of individual tubulin isotypes as well as in arrangement and regulation of intracellular microtubular networks [14]. In GBM cells, βIII-tubulin is a highly studied isotype, mainly due to its differential expression compared to normal mature glia. It is generally acknowledged that higher βIII-tubulin expression occurs in high-grade gliomas and associated with adverse clinical outcomes [15].

Flubendazole (FLU) is a member of the benzimidazole group, a drug commonly used in veterinary and human medicine for its anthelmintic properties. This anthelmintic effect is based on its ability to bind to β-tubulin [16], which causes inhibition of tubulin polymerization leading to microtubule disruption and loss of function [17]. Several studies report promising anticancer effects of FLU in various cancer cells, including breast cancer cells, colon cancer cells, non-small cell lung carcinoma, melanoma cells, and glioma cells [2,18,19,20]. These effects are thought to be mediated not only via microtubule disruption but also via interference with several signaling pathways, including IL/JAK/STAT3, AKT, NF-κB and others, ultimately to cell cycle arrest, autophagy, apoptosis and/or other types of cell death [19,21,22,23]. In addition, in GBM, FLU has been reported to affect G2/M cell cycle progression through the P53/P21/Cyclin B1 pathway [22,24].

In this study, we aimed to investigate the inhibitory effect of TMZ, FLU and their combination on stabilized GBM cell lines with a specific focus on cell cycle modifications and the microtubule cytoskeleton.

## 2. Materials and Methods

### 2.1. Cell Culture

Human glioma cell lines A172 (ATCC© CRL-1620™), T98G (ATCC© CRL-1690™) and U118MG (ATCC© HTB-15™) were purchased from ATCC (Manassas, VA, USA). A172 and U118MG cells were cultivated in cultivation flasks in Dulbecco’s Modified Eagle’s Medium (LGC Standards, Teddington, UK) enriched with 1% penicillin/streptomycin (Thermo Fisher Scientific, Waltham, MA, USA) and 10% fetal bovine serum (Gibco, Thermo Fisher Scientific, Waltham, MA, USA). T98G cells were cultivated in Eagle’s Minimum Essential Medium (LGC Standards, Teddington, UK) supplemented with 1% penicillin/streptomycin (Life Technologies, Thermo Fisher Scientific, Waltham, MA, USA) and 10% fetal bovine serum (Gibco, Thermo Fisher Scientific, Waltham, MA, USA). All cells were cultivated in a cell incubator at 37 °C and 5% CO_2_ and periodically checked for the absence of mycoplasma contaminating the cell cultures.

### 2.2. Viability/Proliferation

Cell viability and proliferation were measured using the WST-1 assay. The cells were seeded in a 96-well plate (7500 cells/well). Subsequently, after 24 h cultivation, TMZ (50 µM, 250 µM, 500 µM), FLU (0.5 µM), or the combinations of drugs were added to cells. After 48 h of the cultivation, 100 µL of WST-1 reagent (diluted 1:20) was used for a further 2 h incubation. The absorbance was measured using a spectrophotometer Tecan Infinite M200 Microplate Reader (Tecan, Männedorf, Switzerland) at 450 nm with reference wavelength 650 nm. All experiments were performed in three independent repetitions.

### 2.3. Determination of Cell Viability by Trypan Blue Exclusion

A Trypan blue exclusion assay was used for measuring cell viability. The cells were treated with TMZ (50 µM, 500 µM), FLU (0.5 µM) or combination of TMZ + FLU (TMZ 50 µM + FLU 0.5 µM, TMZ 500 µM + FLU 0.5 µM) for 48 h. For each condition, floating cells were collected from the culture medium. Adherent cells were trypsinized to detach them from the culture flask and then resuspended in culture medium. The collected floating cells and trypsinized adherent cells were pooled together. A 1:1 mixture of 50 µL of cell suspension and 50 µL of 0.5% Trypan blue solution was prepared. The pooled cell suspension stained with Trypan blue was loaded into a Bürker chamber. The numbers of viable and non-viable cells were counted using a Nikon Eclipse E200 light microscope (Nikon, Tokyo, Japan). The percentage of viable cells was calculated relative to the total number of cells counted.

### 2.4. Phase Contrast Microscopy

The cells A172, T98G and U118MG seeded in tissue culture flasks were treated with TMZ (50 µM, 500 µM), FLU (0.5 µM), and their combinations (TMZ 50 µM + FLU 0.5 µM, TMZ 500 µM + FLU 0.5 µM) and the effect of these drugs on cell viability and morphology was evaluated using Olympus IX70 inverted phase-contrast microscope (Olympus Corporation, Tokyo, Japan) after 24 h, 48 h and 72 h of the treatment.

### 2.5. Apoptosis Luminescent Assay

The cells were seeded in a 96-well plate (7500 cells/well) and treated with TMZ (50 µM, 500 µM), FLU (0.5 µM) or TMZ + FLU (TMZ 50 µM + FLU 0.5 µM, TMZ 500 µM + FLU 0.5 µM) and were harvested after 4 h, 8 h, 16 h and 24 h of treatment using caspase lysis buffer (5 mM DTT, 50 mM HEPES, 5 mM CHAPS). The activity of caspases 3/7, 8 and 9 was measured using Promega Caspase-Glo assay (Promega, Madison, WI, USA) according to the manufacturer’s instructions.

### 2.6. Immunofluorescence

A172, T98G and U118MG were grown in cytospin chambers in the presence of TMZ (50 µM, 500 µM), FLU (0.5 µM) or TMZ + FLU (TMZ 50 µM + FLU 0.5 µM, TMZ 500 µM + FLU 0.5 µM) for 24 h. The cells were fixed for 15 min with ice-cold methanol at −20 °C, followed by 15 min with 4% paraformaldehyde at room temperature and blocked for 1 h with 1% BSA + 0.3% Triton X in 1xPBS at room temperature. Subsequently, the cells were incubated with primary antibodies (diluted by 1% BSA + 0.3% Triton X in 1xPBS) against α-tubulin (1:100, Abcam, Cambridge, UK) and βIII-tubulin (1:50, Cell Signaling Technology, Danvers, MA, USA) at 4 °C overnight. The cells were washed twice with 1xPBS and incubated for 2 h with secondary antibodies Alexa Fluor goat anti-mouse 488 (1:250, Invitrogen, Carlsbad, CA, USA) or Alexa Fluor goat anti-rabbit 488 (1:250, Invitrogen) and further labeled with DAPI (10 µg/mL, Sigma-Aldrich, St. Louis, MO, USA) at room temperature. The cells were washed twice with 1xPBS, mounted using ProLong Gold Antifade Mountant (Molecular Probes, Eugene, OR, USA), and assessed using a fluorescent microscope Nikon Eclipse E400 (Nikon Corporation, Tokyo, Japan).

### 2.7. Isolation of RNA, Synthesis of cDNA, Real-Time RT-PCR

The cells A172, T98G and U118MG were treated with TMZ (50 µM, 500 µM), FLU (0.5 µM) or their combinations (TMZ 50 µM + FLU 0.5 µM, TMZ 500 µM + FLU 0.5 µM) in a 6-well plate (150,000 cells/well) for 24 h and harvested using TRI Reagent^®^ (Sigma-Aldrich, St. Louis, MO, USA). According to the manufacturer’s instructions, total RNA from cells was isolated using Direct-zol RNA Miniprep Kits (Zymo Research, Irvine, CA, USA). RNA concentration and purity were measured using a NanoDrop ND-2000 UV-Vis Spectrophotometer (Thermo Fisher Scientific, Waltham, MA, USA). The absorption ratio A260/A280 for all tested samples was greater than 1.8. First Strand cDNA was prepared from 1 µg of total RNA according to manufacturer’s instructions with First Strand cDNA Synthesis Kit (Thermo Fisher Scientific, Waltham, MA, USA), and obtained cDNA was 5x diluted before qPCR reaction.

All used primers were designed using Primer3 software, version 2.6.1. Their specificity was checked using the NCBI Blast tool. Primers were synthesized by Generi Biotech (Hradec Kralove, Czech Republic). Sequences of primers are provided in the Supplementary Table S1.

The qPCR analysis was performed using SYBR Green I detection with a reaction mixture prepared according to the manufacturer’s instructions with 5 µL of diluted cDNA and primers in a final concentration of 100 nM. TATA-binding protein (TBP) or β2-microglobulin (B2M) was used as the reference gene. All reactions were performed with the LightCycler96 Real-Time PCR Detection System (Roche, Berlin, Germany) with a standard pattern of denaturation (95 °C for 10 min), 40 cycles of amplification (10 s denaturation at 95 °C, 10 s annealing at 60 °C, 10 s extension at 72 °C) and fluorescence data collection using dissociation protocol with gradient from 65 °C to 95 °C. The calculation was performed by the ΔΔCq method, and the expression of the tested markers of treated cells was expressed as a multiple of the expression of markers of control cells. The data were indicated as fold change.

### 2.8. Western Blot Analysis

Non-treated and treated cells were harvested in ice-cold lysis buffer (1% Triton X-100, 10% glycerol, 50 mM Tris/HCl, 150 mM NaCl, 50 mM NaF, 2 mM EDTA, 2 mM EGTA, 10 mM sodium pyrophosphate, 200 µM Na_3_VO_4_, 2 mM DTT, 40 mM β-glycerolphosphate). Obtained cell lysate was homogenized and centrifuged for 10 min (4 °C, 13,000 rpm). The total protein quantity in the prepared supernatant was measured using Pierce™ BCA Protein Assay Kit (Thermo Fisher Scientific, Waltham, MA, USA). Samples were boiled at 95 °C for 5 min, and 30 µg of total protein per well was applied to the polyacrylamide gel. Electrophoresis was performed at a constant voltage of 120 V. The separated proteins were transferred (90 min, 100 V) to a PVDF membrane. The membrane was blocked with 5% nonfat dry milk in TBST buffer for 2 h at room temperature. Incubation of the membrane with primary antibodies (monoclonal rabbit anti-GAPDH (1:1000; Cell Signaling Technology, Danvers, MA, USA); monoclonal rabbit anti-βIII-tubulin (1:1000; Cell Signaling Technology, Danvers, MA, USA); monoclonal rabbit anti-cyclin B1 (1:1000; Cell Signaling Technology, Danvers, MA, USA); polyclonal rabbit anti-cdc2 p34 (1:1000; Santa Cruz Biotechnology, Dallas, TX, USA); monoclonal mouse anti-α-tubulin (1:10,000; Abcam, Cambridge, UK) diluted in 5% BSA or 5% nonfat dry milk in TBST, as recommended by the manufacturer, was performed at 4 °C overnight. Following membrane washing by TBST buffer, membranes were incubated with appropriate secondary HRP-conjugated antibody (diluted in 1% BSA in TBST buffer) for 2 h at room temperature. Protein detection was performed using Amersham™ ECL™ Prime Western Blotting Detection Reagent and Azure c600 Gel Imaging System (Azure Biosystems, Dublin, CA, USA). AzureSpot Analysis Software, version 2.0.062 (Azure Biosystems) was used for relative protein quantification with GAPDH as the reference protein. Original immunoblot images for all Western blot analyses are provided in the Supplementary Materials. Membranes were cut after protein transfer, and each cut was tested with the appropriate primary antibody.

### 2.9. Flow Cytometry—Cell Cycle Distribution

After 24 h exposure to TMZ (50 µM, 500 µM), FLU (0.5 µM), or their combinations (TMZ 50 µM + FLU 0.5 µM and TMZ 500 µM + FLU 0.5 µM), the cells were harvested and fixed in 70% ice-cold ethanol. Fixed samples were stored at 4 °C until further processing. Cells were then pelleted by centrifugation, ethanol was removed, and the cells were washed twice with ice-cold phosphate-buffered saline (PBS). For the detection of low molecular weight DNA fragments, the cells were incubated for 5 min at room temperature in phosphate-citrate buffer (192 mL 0.2 M Na_2_HPO_4_ and 8 mL of 0.1 M citric acid; pH 7.8). Following an additional PBS wash, the cells were stained with propidium iodide in Vindelov’s solution for 1 h at 37 °C. Cellular DNA content was subsequently measured using a CytoFLEX LX Flow Cytometer (Beckman Coulter, Brea, CA, USA) with a 488 nm excitation wavelength, and fluorescence was collected in the 600–620 nm emission range. Cell cycle distribution was evaluated using Kaluza Analysis 2.1 software (Beckman Coulter, USA).

### 2.10. Flow Cytometry—Annexin V/PI Assay for Cell Viability and Apoptosis Analysis

Apoptosis was assessed by flow cytometry using the Alexa Fluor^®^ 488 Annexin V/Dead Cell Apoptosis kit (Life Technologies, Carlsbad, CA, USA) according to the manufacturer’s protocol. This assay is based on the binding of Alexa Fluor^®^ 488-conjugated Annexin V to phosphatidylserine in the presence of Ca^2+^ and on propidium iodide staining of DNA in cells with compromised membrane integrity. At least 20,000 events were recorded for each sample using a CytoFLEX LX Flow Cytometer (Beckman Coulter, USA). The acquired data were processed using Kaluza Analysis 2.1 software (Beckman Coulter, USA).

### 2.11. LC-MS Analysis

The GBM cells A172, T98G and U118MG were cultivated in 6-well plate (150,000 cells/well) and treated with TMZ (50 µM, 500 µM) for 10 min, 30 min, 2 h; FLU (0.5 µM) for 2 h, 4 h, 24 h and their combination for 10 min, 30 min, 2 h, 4 h, 24 h. Afterwards, the cells were collected and resuspended in 500 µL of sterile distilled water. Frozen tissues (brains, tumors) were homogenized in 4 volumes of cold PBS (*w*/*v*) using FastPrep-24™ 5G (MP Biomedicals, Santa Ana, CA, USA) sample disruption instrument. Homogenized tissue samples, cell lysates, or culture media (100 µL) were mixed with an equal volume of methanol/acetonitrile, vortexed for 15 min, and centrifuged at 14,000× *g* for 3 min. The resulting supernatant was passed through a 0.22 µm PTFE syringe filter into the glass sample vial for analysis.

TMZ was quantified using an Agilent 1290 Infinity II UHPLC system coupled to the Agilent 6470 QqQ mass spectrometer (Agilent Technologies, Santa Clara, CA, USA). A sample volume of 1 µL was injected. Separation was performed on the Zorbax Eclipse Plus RRHD C18 column (2.1 × 50 mm, 1.8 µm; Agilent Technologies, Santa Clara, CA, USA) maintained at 30 °C, while the autosampler temperature was set to 15 °C. Gradient elution was carried out at a flow rate of 0.4 mL/min using 0.1% formic acid in water and 0.1% formic acid in methanol as mobile phases. The gradient program was as follows: 0–0.5 min 95:5; 0.5–3 min 5:95; 3–4 min 5:95; 4–5 min 95:5. The MS source parameters were set as follows: drying gas 200 °C at 2 L/min; sheath gas 400 °C at 12 L/min; nebulizer pressure 25 psi; capillary voltage 2500 V; nozzle voltage 0 V. TMZ was detected in the positive ion mode as [M + H]+ ions using the transitions *m*/*z* 195→138 and 55, with a dwell time of 50 ms, fragmentor voltage of 88 V, collision energy of 8 and 28 V, and cell accelerator voltage of 4 V.

FLU was analyzed using the same instrument. Chromatographic separation was performed under the same gradient elution conditions, with a flow rate of 0.4 mL/min and mobile phases consisting of 0.1% formic acid in water and methanol. The autosampler was maintained at 15 °C, and the Zorbax Eclipse Plus RRHD C18 column (2.1 × 50 mm, 1.8 µm; PN 959757-902; Agilent Technologies, Santa Clara, CA, USA) was kept at 30 °C. The MS source parameters were adjusted as follows: drying gas 230 °C at 4 L/min; sheath gas 400 °C at 12 L/min; nebulizer pressure 40 psi; capillary voltage 3000 V; nozzle voltage 0 V. FLU was detected in the positive ion mode as [M + H]+ ions using the transitions *m*/*z* 314→282, 123 and 95, with a dwell time of 30 ms, fragmentor voltage of 128 V, collision energy of 24, 40 and 56 V, and cell accelerator voltage of 4 V.

### 2.12. Preparation of Mice Model

Female Athymic immunodeficient Nude-Foxn1nu mice were purchased from Velaz, s.r.o. (Prague, Czech Republic) for in vivo analysis. The project was approved by the Ministry of Education, Youth and Sports of the Czech Republic (Reference No. MSMT-18525/2021-3), confirming that all methods were carried out in accordance with relevant guidelines and regulations, including ARRIVE guidelines.

The cancer cell line U118MG was used for cell implantation. Three million cells per puncture were implanted on the left and right dorsal sides of athymic nude mice. The condition and weight of the mice and the tumor size were periodically checked. Two weeks after implantation, the mice were divided into six groups and each group received the appropriate tested drug (TMZ, FLU) or their combination via peroral gavage every day. The groups were designed as follows: control group (5 mice), FLU 10 mg/kg (4 mice), FLU 25 mg/kg (4 mice), TMZ 0.9 mg/kg (3 mice), FLU 10 mg/kg + TMZ 0.9 mg/kg (3 mice) and FLU 25 mg/kg + TMZ 0.9 mg/kg (3 mice). TMZ and FLU were diluted in 1% methylcellulose to suitable concentrations.

On the 14th day of treatment, mice were anesthetized with isoflurane and were sacrificed 15 min after drug administration. The tumors and selected organs (heart, liver and brain) were obtained, weighed and preserved until further analysis at −80 °C. Part of the obtained tumor tissue from each group was fixed in formalin and thus prepared tumors for IHC analysis were stored at room temperature. Furthermore, blood was obtained from the posterior vena cava using K_3_EDTA blood collection tube (Sarstedt, Brand-Erbisdorf, Germany) and then centrifuged twice for 5 min at 3000 rpm to separate plasma, which was then stored until further analysis at −80 °C.

For qPCR analysis, tumors were disrupted and homogenized using 200 µL of RiboEx™ (GeneAll Biotechnology, Seoul, Republic of Korea), a metal bead and TissueLyser LT (Qiagen, Hilden, Germany). Total RNA from the tumor was isolated using GeneAll^®^ Hybrid-R™ (GeneAll Biotechnology, Seoul, Republic of Korea) according to the manufacturer’s instructions. Measurement of RNA concentration and purification, First Strand cDNA preparation, and qPCR were performed in the same way as for in vitro analysis.

The tumors for Western blot analysis were disrupted using 100 µL of ice-cold lysis buffer (the same as for in vitro analysis), metal beads and TissueLyser LT (Qiagen, Hilden, Germany). Subsequently, cell lysates were homogenized and centrifuged for 10 min (4 °C, 13,000 rpm). The following procedure was the same as for in vitro analysis. Isolated total protein concentration and further analysis was performed as described above.

### 2.13. Immunohistochemical Analysis

Part of the removed mouse tumor tissue was fixed in 4% buffered formaldehyde solution, embedded in paraffin and processed for routine histopathological assessment. In total, 3 µm thick sections were cut and stained with hematoxylin–eosin to assess the amount of tumor tissue for the analysis. Immunohistochemical detection of proliferation-related antigen Ki67 (MIB1, RTU, Dako, Glostrup, Denmark) was performed on Dako Omnis automated immunostainer (Agilent Technologies, Santa Clara, CA, USA) with Dako EnVision FLEX visualization system (Dako, Glostrup, Denmark), using horseradish peroxidase for visualization of the antibody–antigen reaction and hematoxylin counterstaining. The quantitative evaluation of Ki-67 immunostaining was performed by an experienced pathologist who was completely blinded to the experimental groups (control vs. treated). The samples were randomized and encoded prior to the analysis. For each sample, approximately 20 independent microscopic fields were analyzed to ensure robust and representative scoring.

### 2.14. MGMT Methylation Status

The MGMT promoter methylation status was assessed in FFPE samples. DNA was isolated using the DNA Sample Preparation Kit (Roche, Berlin, Germany), followed by bisulfite conversion with the EZ DNA Methylation-Gold Kit (Zymo Research, Irvine, CA, USA). Hypermethylation of the O^6^-methylguanine-DNA-methyltransferase (MGMT) promoter was then detected and quantified by methylation-specific real-time PCR using the geneMAP MGMT Methylation Analysis Kit (Genmark Saglik Urunleri, Istanbul, Türkiye) with CE-IVD certification.

### 2.15. Statistical Analysis

All statistical analyses were carried out using GraphPad Prism 9.4 Software. Before conducting comparative analyses, the data were tested for normality using the Shapiro–Wilk test, and for homogeneity of variances using the Brown–Forsythe test. Two-way ANOVA and Sidak’s multiple comparison test were used with *p*-value < 0.05 (tagged as * or #) considered statistically significant. IC50 was calculated using nonlinear regression with GraphPad Prism 9.4 Software. The results of experiments are described as the average of at least two measurements.

## 3. Results

### 3.1. The Combination of TMZ + FLU Reduced Cell Proliferation and Induced Morphological Changes

**Figure 1:**
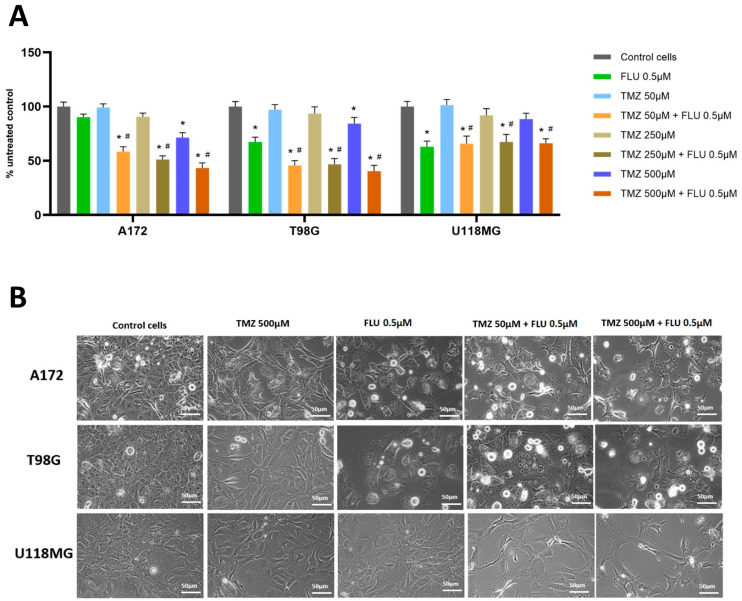
The effect of the combination of TMZ + FLU on cell proliferation and morphological changes in GBM cells. (**A**) Comparison of cell proliferation after the administration of TMZ + FLU combination and TMZ in monotherapy. A172, T98G and U118MG cells were treated with TMZ (50 µM, 250 µM, 500 µM), FLU (0.5 µM) or the combination of these drugs. Cell proliferation was measured after 48 h of treatment using the WST-1 assay. Measurements were performed in three independent experiments. * *p* ˂ 0.05 vs. untreated control, # *p* ˂ 0.05 the combination of TMZ and FLU vs. corresponding TMZ (TMZ 50 µM, 250 µM or 500 µM). (**B**) Microscopic evaluation of cell proliferation and morphological changes in GBM cells. Changes were observed after 48 h treatment with TMZ (50 µM, 500 µM), FLU 0.5 µM and their combination using phase contrast microscopy. Magnification 400×, scale bar 20 µm.

The effect of TMZ, FLU, and their combination was evaluated biochemically (WST-1 assay) and cytometrically (phase microscopy). Three stabilized GBM cell lines (A172, T98G, U118MG) were used in this study. Based on previous testing of FLU efficacy, concentration of 0.5 µM FLU was selected for further experiments. Cells were treated for 48 h with TMZ (50 µM, 250 µM, 500 µM), FLU (0.5 µM) or their combination. The combination of TMZ and FLU decreased cell proliferation in all tested cell lines. In all tested cells the co-administration of TMZ + FLU significantly reduced cell proliferation compared with TMZ monotherapy (Figure 1A). Based on these results, TMZ 50 µM, TMZ 500 µM and FLU 0.5 µM (or their combination) were selected for further analyses.

The phase contrast microscopy showed that TMZ + FLU treatment visibly reduced the density of glioma cells and induced changes in cell morphology (Figure 1B). In addition to changes in cell shape and size, multinuclear cells and membrane blebs were observed. The lower cell density was consistent with the reduced proliferation detected by the WST-1 assay. However, Trypan blue exclusion staining did not indicate a significant reduction in viability of GBM cell lines after 48 h exposure to the combination of TMZ (50 or 500 µM) and FLU (0.5 µM) (Supplementary Figure S1).

Moreover, GBM cells showed higher sensitivity to FLU administration than TMZ treatment. While the half-maximal inhibitory concentration (IC_50_) of FLU ranged from 2.061 µM to 6.530 µM, the IC_50_ of TMZ reached significantly higher values (from 1709.0 µM to 2567.0 µM) (Table 1). Since differences in TMZ sensitivity may be related to MGMT promoter methylation status of GBM cells, MGMT methylation status was examined in all tested cell lines. Surprisingly, A172 and T98G showed MGMT promoter methylation, whereas U118MG cell had an unmethylated MGMT promoter.

### 3.2. TMZ + FLU Co-Treatment Was Associated with Caspase Activation

**Figure 2:**
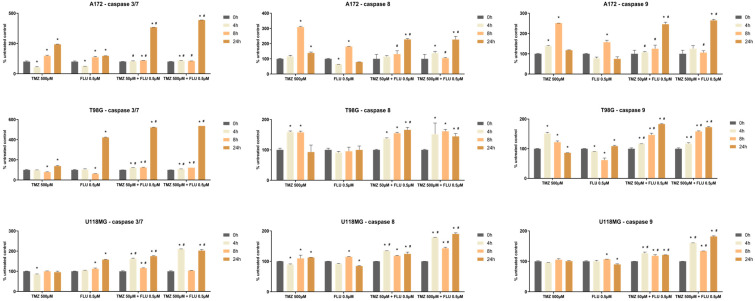
The effect of TMZ + FLU combination on the activity of caspases 3/7, 8 and 9 in GBM cells (A172, T98G, and U118MG). After treatment with TMZ 500 µM, FLU 0.5 µM, TMZ 50 µM + FLU 0.5 µM or TMZ 500 µM + FLU 0.5 µM for 4 h, 8 h and 24 h, the activity of caspases was measured. * *p* ˂ 0.05 vs. untreated control, # *p* ˂ 0.05 the combination of TMZ and FLU vs. corresponding TMZ (TMZ 50 µM or 500 µM).

Subsequently, the effect of TMZ and FLU administration in monotherapy and combination on caspase activity was compared. Cells were exposed to TMZ, FLU or TMZ + FLU for 4 h, 8 h, and 24 h. In all tested cell lines, the drug combination was associated with increased activity of initiator caspases 8, 9 and effector caspases 3/7 (Figure 2). Furthermore, caspase activity in TMZ + FLU-treated cells was generally higher than in cells treated with TMZ monotherapy, particularly after the longer incubation time (24 h).

The ability of TMZ and FLU co-treatment to induce cell death and apoptosis was determined by flow cytometry 48 h after treatment. No significant changes in early or late apoptosis were observed after the combined TMZ + FLU treatment in A172, T98G, and U118MG cells compared with TMZ in monotherapy. Neverthelles, reduced cell viability following combined treatment was observed in tested cell lines, as shown in Supplementary Figure S2.

### 3.3. The Effect of the Combination of TMZ + FLU on G2/M Cell Cycle Accumulation

**Figure 3:**
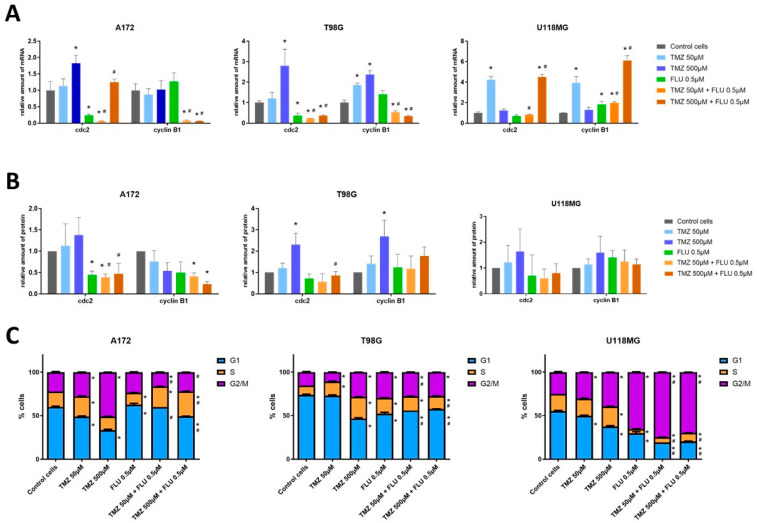
The effect of the combination of TMZ + FLU on the expression of selected cell cycle markers and cell cycle distribution. The cells were treated with TMZ (50 µM and 500 µM), FLU (0.5 µM) or their combinations (TMZ 50 µM + FLU 0.5 µM and TMZ 500 µM + FLU 0.5 µM) for 24 h for analysis using flow cytometry and RT-PCR analysis and 48 h for Western blot analysis. (**A**) Changes in cdc2 and cyclin B1 expression at the mRNA level in GBM cells were performed using RT-PCR. TBP was used as a reference gene. (**B**) Changes in the expression of cdc2 and cyclin B1 at the protein level were assessed by chemiluminescence evaluation of results from Western blot analysis as a percentage of control cells. GAPDH was used as a reference protein. (**C**) The relative distribution of glioma cells at different cell cycle phases after drug treatment was measured using flow cytometry. * *p* ˂ 0.05 vs. untreated control, # *p* ˂ 0.05 the combination of TMZ and FLU vs. corresponding TMZ (TMZ 50 µM or 500 µM).

Additionally, the distribution of cells in different cell cycle phases was analyzed. Given the previously reported, FLU affect the G2/M phase of the cell cycle, the mRNA and protein expression of cdc2 and cyclin B1 was also evaluated in treated glioma cells. A reduction in cdc2 and cyclin B1 expression was observed at the mRNA level in A172 and T98G cells (Figure 3A). In A172 cells, the combined TMZ + FLU treatment significantly decreased cdc2 and cyclin B1 expression at the protein level, as shown in Figure 3B. However, this effect was less consistent in other tested cell lines (T98G and U118MG), which prompted further analysis of DNA-content distribution by flow cytometry. Flow cytometry analysis showed changes in the distribution of cells across the G1, S and G2/M phase following treatment (Figure 3C). An increased proportion of cells in G2/M phase was observed after TMZ + FLU treatment, particularly in U118MG cells, where the percentage of the G2/M phase cells increased to approximately 70%. In all tested glioma cell lines, especially in T98G cells, an additional population separated from the G1 phase was also observed. However, this observation should be interpreted cautiously, as PI-based DNA-content analysis alone does not allow definitive discrimination between cell cycle changes and apoptotic cell death. Detailed flow cytometry histograms and quantitative percentages of cells in each cell cycle phase are provided in Supplementary Figure S3 and Supplementary Table S2.

### 3.4. Co-Administering TMZ and FLU Altered Microtubule Structure and Reduced Expression of α-Tubulin and βIII-Tubulin

**Figure 4:**
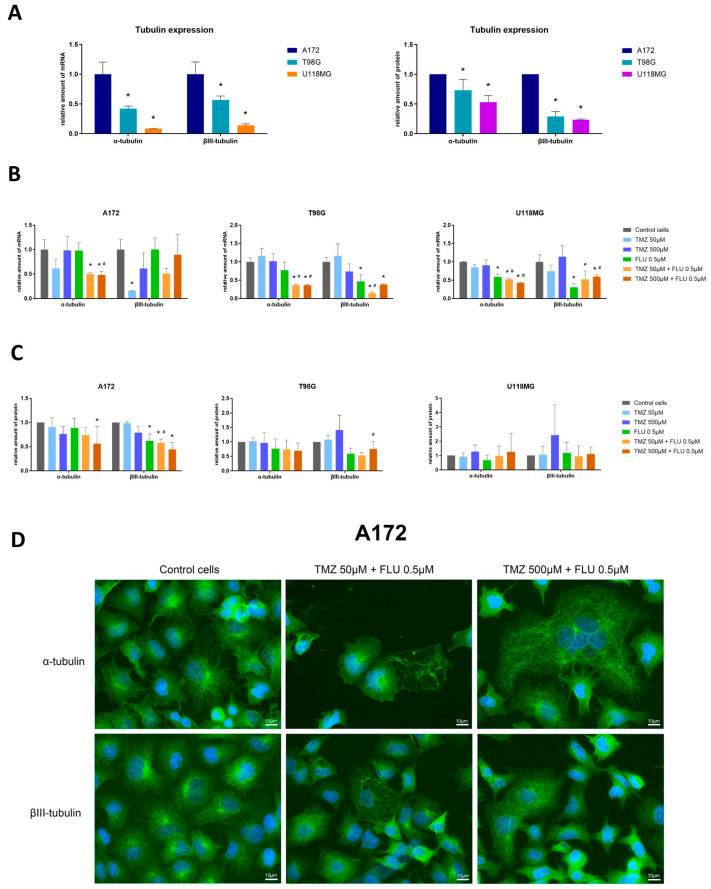
α- and βIII-tubulin expression and microtubule organization in A172, T98G and U118MG. (**A**) The constitutive expression of α- and βIII-tubulin in A172, T98G and U118MG cells was determined at the mRNA and protein level. TBP was used as a reference gene for RT-PCR analysis, while GAPDH was used as a reference protein for Western blot analysis. (**B**) Changes in α-tubulin and βIII-tubulin expression at the mRNA level (after 24 h treatment) in glioma cells were assessed using RT-PCR. TBP was used as a reference gene. (**C**) The expression of α- and βIII-tubulin at the protein level was evaluated in GBM cells after 48 h of treatment using chemiluminescence evaluation of the results from Western blot analysis. The results were expressed as a percentage of control cells, with GAPDH as a reference protein. (**D**) Changes in microtubule organization were observed in A172 cells exposed to the tested drugs for 24 h using fluorescence microscopy (magnification 600×, scale bar 10 µm). * *p* ˂ 0.05 vs. untreated control, # *p* ˂ 0.05 the combination of TMZ and FLU vs. corresponding TMZ (TMZ 50 µM or 500 µM).

Further investigation focused on changes in cellular microtubules, which are among the known targets of FLU. First, the expression of α- and βIII-tubulin was compared in A172, T98G, and U118MG cells at the mRNA and protein levels. The expression of both tubulins differed among the tested cells (Figure 4A). A172 cells showed the highest tubulin expression, whereas U118MG cells exhibited the lowest expression.

Subsequently, the glioma cell lines A172, T98G and U118MG were treated with TMZ (50 µM and 500 µM), FLU 0.5 µM and their combinations (TMZ 50 µM + FLU 0.5 µM and TMZ 500 µM + FLU 0.5 µM). The mRNA and protein levels of α-tubulin and βIII-tubulin were measured to assess changes in their expression. FLU-containing treatments, including TMZ + FLU combinations, were associated with reduced expression of α-tubulin at the mRNA level in all tested cells (Figure 4B). Furthermore, in T98G and U118MG cells, the drug combination was also more effective in reducing βIII-tubulin expression at the mRNA level than the TMZ used in monotherapy. At the protein level, reduced expression of the tested tubulins after the TMZ + FLU treatment was observed mainly in A172 cells (Figure 4C).

Based on these findings, the effect of TMZ + FLU combination on microtubule structure was examined using fluorescence microscopy. TMZ + FLU treatment was associated with visible changes in microtubule organization and in the structure of α- and βIII-tubulin filaments (Figure 4D). Representative images are shown for A172 cells, in which the most pronounced treatment-associated changes were observed.

### 3.5. TMZ + FLU Combination Therapy Affected the Accumulation of TMZ and FLU in GBM Cells

**Figure 5:**
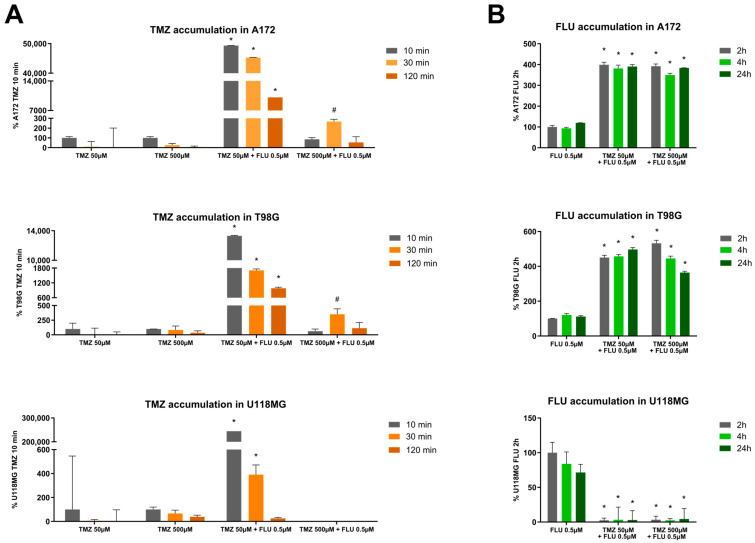
TMZ and FLU accumulation in A172, T98G and U118MG cells after TMZ-, FLU- or TMZ + FLU-treatment. (**A**) The comparison of TMZ accumulation in GBM cells after 10 min, 30 min and 120 min treatment with TMZ 50 µM, TMZ 500 µM, TMZ 50 µM + FLU 0.5 µM and TMZ 500 µM + FLU 0.5 µM. * *p* ˂ 0.05 vs. TMZ 50 µM 10 min; # *p* ˂ 0.05 vs. TMZ 500 µM 10 min. (**B**) The comparison of FLU accumulation in A172, T98G and U118MG cells 2 h, 4 h and 24 h after treatment with FLU 0.5 µM, TMZ 50 µM + FLU 0.5 µM and TMZ 500 µM + FLU 0.5 µM. * *p* ˂ 0.05 vs. FLU 0.5 µM 2 h.

Next, the TMZ and FLU accumulation in glioma cells was investigated. LC/MS analysis was used to compare the intracellular concentrations of both drugs after administration in monotherapy or in combination. TMZ accumulation was increased in all tested glioma cells after TMZ + FLU combination treatment compared with TMZ monotherapy (Figure 5A). In addition, increased FLU accumulation after combination treatment was observed in A172 and T98G cells (Figure 5B). The corresponding absolute mean concentrations of TMZ and FLU determined by LC/MS analysis are provided in Supplementary Table S3.

### 3.6. Co-Administering TMZ and FLU Decreased the Average Tumor Weight and Expression of Ki-67

The effect of treatments on average tumor weight and Ki-67 proliferation index was investigated in the tumors obtained from athymic Foxn1nu mice. These mice were treated p.o. with FLU 10 mg/kg, FLU 25 mg/kg, TMZ 0.9 mg/kg, TMZ 0.9 mg/kg + FLU 10 mg/kg or TMZ 0.9 mg/kg + FLU 25 mg/kg daily for 14 days. Tumor weight was reduced in the TMZ + FLU-treated groups compared with the control group. Ki-67 levels were also lower in the tumors obtained from mice treated with TMZ + FLU than in tumors from mice treated with TMZ monotherapy (Table 2). However, the present data do not demonstrate clear superiority of the combination treatment over FLU monotherapy.

### 3.7. The Combination of TMZ + FLU Was Associated with Changes in α- and βIII-Tubulin Expression In Vivo

**Figure 6:**
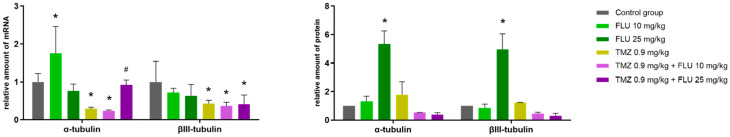
The effect of administration of TMZ, FLU and their combination on expression of α-tubulin and βIII-tubulin in tumors of Nude-Foxn1nu mice. α- and βIII-tubulin expression was determined at the mRNA and protein levels. B2M was used as a reference gene for RT-PCR analysis, while GAPDH was used as a reference protein for Western blot analysis. * *p* ˂ 0.05 vs. untreated control group, # *p* ˂ 0.05 the combinations of TMZ and FLU vs. TMZ 0.9 mg/kg.

The effect of the above-mentioned treatments on α- and βIII-tubulin expression was also analyzed in tumors obtained from Nude-Foxn1nu mice. Tumors from untreated mice were included as controls. Evaluation and comparison of tubulin expression was carried out at the mRNA and protein levels. At the mRNA level, TMZ + FLU co-treatment was associated with reduced expression of both tubulins in selected treatment groups. At the protein level, the response was less consistent, with FLU 25 mg/kg showing increased tubulin expression, whereas TMZ + FLU-treated groups showed lower protein levels compared with FLU 25 mg/kg treatment alone (Figure 6).

### 3.8. The Combination of TMZ + FLU Affected TMZ and FLU Accumulation in the Tumors and Brains of Nu-Nu Mice

**Figure 7:**
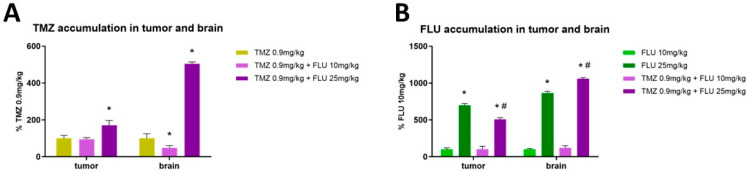
TMZ and FLU accumulation in brains and tumors of Nude-Foxn1nu mice treated with TMZ, FLU or TMZ + FLU. (**A**) The comparison of accumulation of TMZ in tumors and brains of nu-nu mice after treatment with TMZ 0.9 mg/kg, TMZ 0.9 mg/kg + FLU 10 mg/kg and TMZ 0.9 mg/kg + FLU 25 mg/kg. * *p* ˂ 0.05 vs. TMZ 0.9 mg/kg. (**B**) The comparison of FLU accumulation in tumors and brains of nu-nu mice after treatment with FLU 10 mg/kg, FLU 25 mg/kg, TMZ 0.9 mg/kg + FLU 10 mg/kg and TMZ 0.9 mg/kg + FLU 25 mg/kg. * *p* ˂ 0.05 vs. FLU 10 mg/kg; # *p* ˂ 0.05 vs. FLU 25 mg/kg.

Finally, the accumulation of TMZ and FLU in the brains and tumors of Nude-Foxn1nu mice was investigated. Mice were divided into treatment groups as described above. LC/MS analysis was used to compare the accumulation of drugs in the tested tissues after monotherapy and combined treatment. TMZ + FLU co-treatment affected the accumulation of both drugs in tumor and brain tissues. The most pronounced increase in TMZ accumulation was observed after co-administration with FLU 25 mg/kg, particularly in brain tissue. FLU accumulation was also altered by co-administration with TMZ, with increased levels observed mainly in the TMZ 0.9 mg/kg + FLU 25 mg/kg group (Figure 7).

## 4. Discussion

GBM represents a type of malignancy with considerable genotypic and phenotypic heterogeneity which in turn determines its aggressive behavior, chemo and radio resistance and a resulting short-term survival of patients [25]. The limited effectiveness of the standard chemotherapeutic drug temozolomide (TMZ) has prompted various experimental efforts aimed at finding more optimal drug-based treatment strategies for GBM management including the use of combined chemotherapy regimens. This approach has been successfully implemented into treatment of many solid malignancies; however, in the case of GBM, it remains challenging for instance due to the unique brain tissue structure and restricted access to it as well as the versatility and chemoresistance of tumor cells [26].

In our work we have compared inhibitory effects of TMZ, FLU and their combination on several GBM cell lines with individual genotypic background (particularly MGMT status). The rationale for testing FLU alone or in combination with TMZ was based on its known pharmacological and safety profiles and previously ascertained effectiveness towards various cancer cells (e.g., melanoma, leukemic, myeloma, colon and breast cancer cells) [20,27,28,29] including glioma cells [2]. In addition, previous studies have suggested that FLU may reach brain tissue and accumulate in the tumor cells, while being less affected by selected drug efflux mechanisms [22]. These properties make FLU an interesting candidate for further preclinical evaluation in GBM models, although its pharmacokinetic behavior and safety in the context of brain tumor therapy require further investigation.

In the tested panel of cell lines, all of them (i.e., A172, T98G and U118MG) showed higher sensitivity to FLU than to TMZ, as indicated by the determined IC_50_ values (Table 1). This observation was also consistent with the morphological appearance of treated cells (Figure 1B). In addition, the co-administration of TMZ and FLU further enhanced the antiproliferative effect towards treated cells compared with untreated control and TMZ monotherapy. However, the present data do not demonstrate clear superiority of the combination over FLU alone. Morphological analysis, reduced cell density, membrane blebbing, and multinucleation may indicate activation of cell death-related processes. This conclusion might be based on robust and statistically significant activation of both initiator (caspase 8, 9) and effector caspases (caspase 3/7) in exposed cells, namely at 24 h interval (Figure 2). Still, these observations did not translate into a statistically significant increase in late-stage apoptotic populations via Annexin V/PI staining at 48 h (Supplementary Figure S2). This temporal divergence suggests that while the apoptotic enzymatic process was acutely activated by the drug combination, the final execution of classic apoptosis may have been delayed or overridden by alternative cell death pathways. Given that benzimidazoles like FLU induce massive mitotic arrest, cells could have been undergoing mitotic catastrophe or slow senescence—modalities where caspase activation occurs but characteristic membrane phosphatidylserine externalization is less pronounced at standard evaluation time points.

One of the important biomarkers related to GBM prognosis and response to TMZ treatment is promoter methylation of O^6^-methylguanine-DNA-methyltransferase (MGMT) [19]. MGMT promoter methylation is therefore used in stratification of patients in relation to the resistance of cancer cells to TMZ. In our case, A172 and T98G cells (methylated MGMT promoter) as well as U118MG cells (non-methylated MGMT promoter) showed similar sensitivity to combined TMZ + FLU treatment regardless of their MGMT promoter status. This observation suggests that the response to TMZ + FLU co-treatment was not strictly dependent on MGMT promoter methylation status in the tested cell lines. However, further studies are needed to clarify whether FLU modulates TMZ response through MGMT-dependent or MGMT-independent mechanisms.

One possible mechanism may involve the known interaction of FLU with β-tubulin, which can interfere with tubulin polymerization, alter microtubule organization, and affect cell cycle progression in exposed cells [7]. Glioma cells are known to have a rich microtubular network with aberrant expression of βIII-tubulin [20] and heterogeneous expression was also observed for α-tubulin in our model cell lines. In this context, the combination of TMZ and FLU decreased α-tubulin and βIII-tubulin expression at the mRNA level in all tested cell lines. Conversely, decreased expression of α- and βIII-tubulin at the protein level was observed only in A172 cells. This observation could be related to the basal levels of individual tubulins since A172 cells displayed the highest expression of the investigated tubulins compared with the other tested cell lines. Cellular analysis via fluorescence microscopy provided qualitative indices of characteristic morphological changes in treated cells including the formation of multinucleated cells and overall change in cell shape associated with disruption of tubulin fiber structure. However, these immunofluorescence data should be interpreted as supportive and descriptive, since quantitative assessment of microtubule disruption, cell area or filament organization was not performed in the present study. In addition, microtubule-specific changes induced by FLU could potentially modulate TMZ-dependent effects through interference with DNA damage repair processes [21]. Although not addressed in this work, it has recently been suggested that disruption of the microtubular network may disturb the transport of specific proteins involved in DNA repair to their site of activity (i.e., the nucleus), thereby increasing the impact of DNA-damaging agents. This possibility remains speculative in the context of our data and should be addressed in future work.

Microtubule-targeting agents, as well as DNA-damaging agents such as FLU and TMZ, often induce changes in cell cycle progression [2,22,30,31]. Accordingly, co-treatment with TMZ and FLU increased the percentage of cells in the G2/M phase, which was accompanied by changes in cdc2 and cyclin B1 expression. These findings suggest that the antiproliferative effect of combination treatment in GBM cells may be partly associated with G2/M cell cycle accumulation. One of the biological factors that influences the sensitivity of cells to anticancer therapy is p53, a well-established tumor suppressor that is frequently mutated in human cancers. In the absence of p53, or when p53 is not transcriptionally active, the G1 checkpoint control is compromised, causing cells to rely more heavily on the G2/M checkpoint for DNA repair before proceeding to mitosis [32,33]. Interestingly, the observation that the combination of TMZ and FLU resulted in the highest G2/M-phase accumulation in p53-mutated U118MG cells [34] is consistent with the above studies. In this context, the more prominent G2/M accumulation observed in U118MG cells after combined TMZ and FLU treatment may be related to impaired p53-p21 axis signaling.

The effectiveness of many individual anticancer drugs or their combinations depends on their ability to enter the target cell and reach biologically effective levels. This is particularly important in GBM, where the malignancy develops within the restricted environment of brain tissue. Our results showed a higher accumulation of TMZ in all tested cell lines after co-administration of TMZ and FLU compared with TMZ monotherapy. The exact mechanism by which FLU may affect TMZ uptake in the tumor cells is not known. However, it has been suggested that certain benzimidazoles, including FLU, can modulate selected drug transporters such as P-glycoprotein (P-gp), either by acting as direct P-gp inhibitors or by overcoming P-gp-mediated drug resistance in various cancer cell lines [35]. If FLU modulates efflux transporter activity, this could lead to increased intracellular accumulation of TMZ, which is a substrate for certain ABC transporters, including P-gp and BCRP (ABCG2), which normally limit TMZ brain penetration and intracellular concentration [36]. By inhibiting these efflux pumps, FLU could enhance TMZ’s intracellular levels within tumor cells. In addition, P-gp at the blood–brain barrier acts as a significant efflux pump and FLU-dependent modulation of P-gp at this interface could increase TMZ’s brain permeability, leading to higher TMZ concentrations within the central nervous system (CNS) tumor and surrounding brain tissue. Equally, TMZ may influence select drug transporting systems for FLU uptake as indicated by our observation that drug co-administration also facilitated FLU entry into A172 and T98G cells. Despite the attractiveness of this possible FLU-mediated drug transporter modulation mechanism, it remains hypothetical at this point and should be further explored in future studies using direct efflux system assays.

Overall, the tested combination of TMZ and FLU showed biological activity in the GBM cell lines with different MGMT and p53 status. This was reflected by reduced cell proliferation, treatment-associated changes in microtubule-related markers, and demonstrated in terms of mutually reinforced target activity of individual tested compounds and their combinations along with an increased individual compound accumulation in the cells both in vitro as well as in vivo. Interestingly, our in vivo findings revealed that all treatment regimens significantly reduced average tumor weight compared to controls. However, the reduction in tumor mass observed after the combination of TMZ and FLU was comparable to that achieved with FLU 10 mg/kg monotherapy alone. This lack of a further reduction in tumor weight during co-administration could be attributed to several factors. First, FLU at 10 mg/kg exhibits profound monotherapeutic efficacy in this peripheral subcutaneous model, potentially reaching a therapeutic ceiling that masks additive benefits on mass reduction. Second, complex pharmacokinetic or spatial distribution constraints in the tumor microenvironment may limit the structural alignment of DNA damage and microtubule disruption concurrently in vivo. Nonetheless, the combination regimen consistently achieved suppressed proliferation as seen with presence of Ki-67 marker (15%) and altered tissue accumulation of both compounds in brain and tumor tissue. These findings support further preclinical investigation of this combination, while its pharmacological interaction, safety, and therapeutic relevance require additional validation.

The findings of this study should be interpreted in light of several limitations. These include variable responses of the used GBM cell lines to the tested compounds and their combinations, likely reflecting the known genotypic and phenotypic heterogeneity of this malignancy. In particular, lack of clear co-administration benefit over FLU monotherapy in U118MG cells may be related to several factors, including MGMT promoter methylation status, differential expression and dynamics of microtubules and microtubule-associated proteins as well as other factors including cellular transport mechanisms and the activity of relevant signaling pathways. Therefore, further validation in additional models, including primary GBM cell cultures and different culture conditions, would be necessary to confirm the present findings. More clinically relevant models may also help to clarify the mechanism of action of the tested drug combination, particularly with respect to its effect on the microtubular network, DNA repair pathways and intracellular transport systems. Moreover, although the current study was not focused on toxicological aspects of GBM treatment, the absence of normal control cell lines (i.e., astrocytes) should be acknowledged, as such models would provide a better assessment of treatment selectivity and potential side effects. Accordingly, TMZ and FLU concentrations used in our experiments should be interpreted primarily as preclinical experimental conditions rather than as direct equivalents of clinically achievable brain exposures. Possible safety concerns related to brain accumulation and potential toxicity must also be mentioned as pending for future studies, especially because a full pharmacokinetic and toxicity assessment was not performed in the present study.

Finally, the in vivo part of the study was limited by the ability of the employed cell lines to form in vivo tumors, the subcutaneous rather than intracranial tumor localization, and the extent of the performed analyses. In this context, further testing should focus on a more detailed characterization of the pharmacokinetic profiles of combined drug dosing such as the dynamics of drug entry into the CNS and tumors, mutual drug interaction as well as possible accumulation-related CNS toxicity.

## 5. Conclusions

In conclusion, the present study showed that the co-administration of TMZ and FLU reduced proliferation in GBM cell lines regardless of their MGMT promoter methylation and p53 status. However, the current data do not demonstrate classic drug synergy or clear superiority of the combination over FLU monotherapy. Rather, the data suggest an asymmetric cooperative relationship where FLU acts as a highly potent driver of cytotoxicity, and its co-presence substantially augments intracellular TMZ accumulation in vitro and tissue delivery in vivo. This combined therapeutic approach was further associated with reduced cell viability, morphological changes, activation of caspases, and an accumulation of cells in the G2/M phase of the cell cycle. Furthermore, the co-administering TMZ and FLU was associated with changes in the expression of α- and βIII-tubulin, disruption of the microtubule network, and increased intracellular accumulation of both drugs. These findings suggest that FLU may modulate the cellular response of GBM cells to TMZ through several mechanisms, including modulation of microtubule dynamics, interference with DNA damage response pathway, and potential effects on selected drug transporter systems. Further studies are required to clarify the pharmacological interaction between TMZ and FLU and to evaluate the safety and relevance of this combination in more clinically representative GBM models.

## Figures and Tables

**Table 1:** The IC_50_ [µM] values for TMZ and FLU treatment in GBM cells (A172, T98G and U118MG). Cell proliferation was measured after 48 h of treatment using WST-1. The IC_50_ values were calculated using GraphPad Prism 9.4.

	IC_50_ [µM]	
	TMZ	FLU
A172	2567.0	6.530
T98G	2714.0	3.480
U118MG	1709.0	2.061

**Table 2:** Comparison of tumor growth and expression of proliferation marker Ki-67 in tumors after implantation of U118MG cells followed by TMZ, FLU and their combination treatment. The administration of drugs begins from day 15th to day 28th. Tumors were collected 15 min after last application. Confidence interval values of tumor size are shown as mean ± SD. The Ki-67 levels are expressed as the percentage of dividing cells. * *p* ˂ 0.05 vs. untreated control group.

	Average Weight [g]	Ki-67 [%]
Control	0.13 ± 0.02	25
FLU 10 mg/kg	0.03 * ± 0.01	10 *
FLU 25 mg/kg	0.05 * ± 0.02	20
TMZ 0.9 mg/kg	0.05 * ± 0.03	20
TMZ 0.9 mg/kg + FLU 10 mg/kg	0.06 * ± 0.02	15 *
TMZ 0.9 mg/kg + FLU 25 mg/kg	0.07 * ± 0.01	15 *

## Data Availability

The raw data supporting the conclusions of this article will be made available by the authors on request.

## Supplementary Materials

The following supporting information can be downloaded at: https://www.mdpi.com/article/10.3390/cells15141239/s1, Figure S1: The effects of TMZ (50 or 500 µM) and FLU (0.5 µM), alone and in combination, on viability of GBM cells A172, T98G and U118MG; Figure S2: The effects of TMZ (50 or 500 µM) and FLU (0.5 µM), alone and in combination, on apoptosis of GBM cells A172, T98G and U118MG; Figure S3: Representative PI flow cytometry histograms used for cell-cycle analysis of A172, T98G and U118MG cells following TMZ, FLU and combined treatment; Figure S4: Representative images from immunohistochemical analysis of Ki-67 expression in tumors collected from treated athymic nude mice with implanted U118MG glioma cells; Table S1: Sequences of primers used in the study; Table S2: Cell-cycle distribution [%] of GBM cells after treatment with TMZ, FLU and their combination; Table S3: Absolute concentrations of TMZ and FLU in GBM cells determined by LC-MS analysis.
