# Supplementary material for: The Effect of the Combination of Temozolomide and Flubendazole on Glioblastoma Cells
Source: Cells. 2026 Jul 9;15(14):1239. doi: 10.3390/cells15141239 (PMC13406143; doi:10.3390/cells15141239)
Supplement: Supplementary file 1 [file cells-15-01239-s001.zip › Supplementary material.pdf]

SUPPLEMENTARY MATERIAL

Supplementary Table S1: Sequences of primers used in the study

cdc2_F	AGTCAGTCTTCAGGATGTGCT
cdc2_R	GAAGAATCCATGTACTGACCAGG
cyclin B1_F	TGATACTGCCTCTCCAAGCC
cyclin B1_R	CTGCATCCACATCATTTACTGC
α-tubulin_F	ACATCGACCGCCTAAGAGTC
α-tubulin_R	CGAAGAGGAGAGGTTGTTGC
βIII-tubulin_F	ATTCTGGTGGACCTGGAACC
βIII-tubulin_R	CCCACTCTGACCAAAGATGAA
B2M_F	TGCTGTCTCCATGTTTGATGTATC
B2M_R	TCTCTGCTCCCCACCTCTAAG
TBP_F	TGGCGTGTGAAGATAACCCAA
TBP_R	TGGCAAACCAGAAACCCTTG

Supplementary Table S2: Cell-cycle distribution [%] of GBM cells after treatment with TMZ, FLU and their combination

Cell line	Treatment	G1 [%]	S [%]	G2/M [%]
**A172**	Control cells	60.05	17.63	22.31
	TMZ 50 μM	48.88	23.25	27.87
	TMZ 500 μM	33.25	15.76	50.99
	FLU 0.5 μM	62.56	13.77	23.68
	TMZ 50 μM + FLU 0.5 μM	59.95	23.67	16.38
	TMZ 500 μM + FLU 0.5 μM	49.22	28.69	22.09
**T98G**	Control cells	73.86	10.72	15.42
	TMZ 50 μM	72.99	16.19	10.82
	TMZ 500 μM	46.50	25.04	28.46
	FLU 0.5 μM	52.19	18.05	29.76
	TMZ 50 μM + FLU 0.5 μM	55.84	16.35	27.81
	TMZ 500 μM + FLU 0.5 μM	57.60	14.82	27.59
**U118MG**	Control cells	55.23	19.72	25.04
	TMZ 50 μM	49.70	19.77	30.53
	TMZ 500 μM	37.33	23.13	39.54
	FLU 0.5 μM	29.77	4.71	65.52
	TMZ 50 μM + FLU 0.5 μM	19.28	5.74	74.98
	TMZ 500 μM + FLU 0.5 μM	20.42	9.60	69.98

Supplementary Table S3: Absolute concentrations of TMZ and FLU in GBM cells determined by LC-MS analysis.

Cell line	Treatment	Mean TMZ [ng/mL]			Mean FLU [pg/mL]		
		**10 min**	**30 min**	**120 min**	**2 h**	**4 h**	**24 h**
**A172**	TMZ 50 μM	0.009	0.001	<0.001	N/A	N/A	N/A
	TMZ 500 μM	22.370	5.955	1.421	N/A	N/A	N/A
	FLU 0.5 μM	N/A	N/A	N/A	1026.0	960.0	1227.6
	TMZ 50 μM + FLU 0.5 μM	4.239	3.892	19.039	4092.9	3908.5	4004.0
	TMZ 500 μM + FLU 0.5 μM	19.039	59.693	12.144	4020.2	3589.6	3935.7
**T98G**	TMZ 50 μM	0.030	<0.001	<0.001	N/A	N/A	N/A
	TMZ 500 μM	15.729	13.150	5.573	N/A	N/A	N/A
	FLU 0.5 μM	N/A	N/A	N/A	1041.7	1259.7	1162.9
	TMZ 50 μM + FLU 0.5 μM	3.976	0.509	0.293	4693.6	4764.8	5173.6
	TMZ 500 μM + FLU 0.5 μM	9.739	55.113	17.946	5542.0	4633.1	3795.7
**U118MG**	TMZ 50 μM	<0.001	<0.001	<0.001	N/A	N/A	N/A
	TMZ 500 μM	67.428	44.687	26.064	N/A	N/A	N/A
	FLU 0.5 μM	N/A	N/A	N/A	852.6	714.1	609.0
	TMZ 50 μM + FLU 0.5 μM	0.2863	<0.001	<0.001	22.3	27.1	24.3
	TMZ 500 μM + FLU 0.5 μM	<0.001	<0.001	<0.001	28.5	20.6	36.9

Values represent mean absolute concentrations of TMZ and FLU determined by LC-MS analysis. Values are rounded to three decimal places. Values reported as <0.001 indicate very low calculated concentrations below the displayed decimal precision and should not be interpreted as complete absence of the compound. N/A, not applicable.

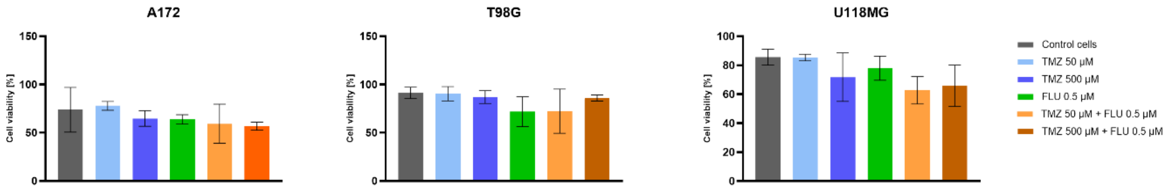

Supplementary Figure S1 The effects of TMZ (50 or 500 μM) and FLU (0.5 μM), alone and in combination, on viability of GBM cells A172, T98G and U118MG. Cell viability was determined at 48 h after treatment using the Trypan blue vital dye exclusion test. Data are shown as mean values ± SD of at least four independent experiments. *$p < 0.05$ vs. untreated control.

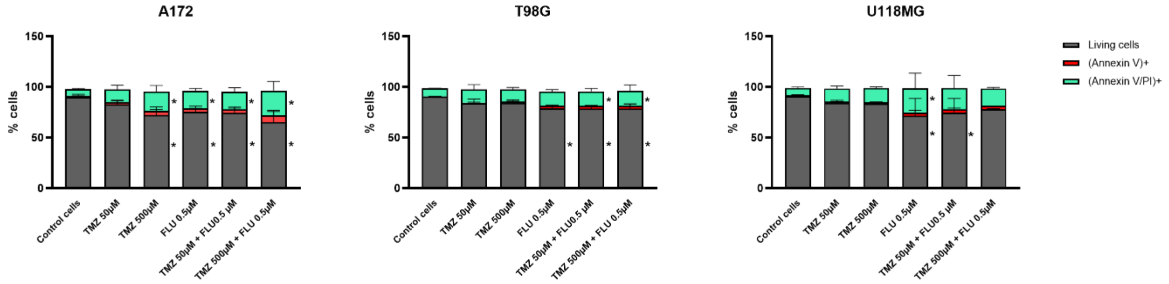

Supplementary Figure S2 The effects of TMZ (50 or 500 μM) and FLU (0.5 μM), alone and in combination, on apoptosis of GBM cells A172, T98G and U118MG. The bar graph represents the percentage of living cells, early (Annexin V+) and late (Annexin V/PI+) apoptotic cells 48 h following exposure. The results are shown as the mean ± SD from three experiments. *$p < 0.05$ vs. untreated control.

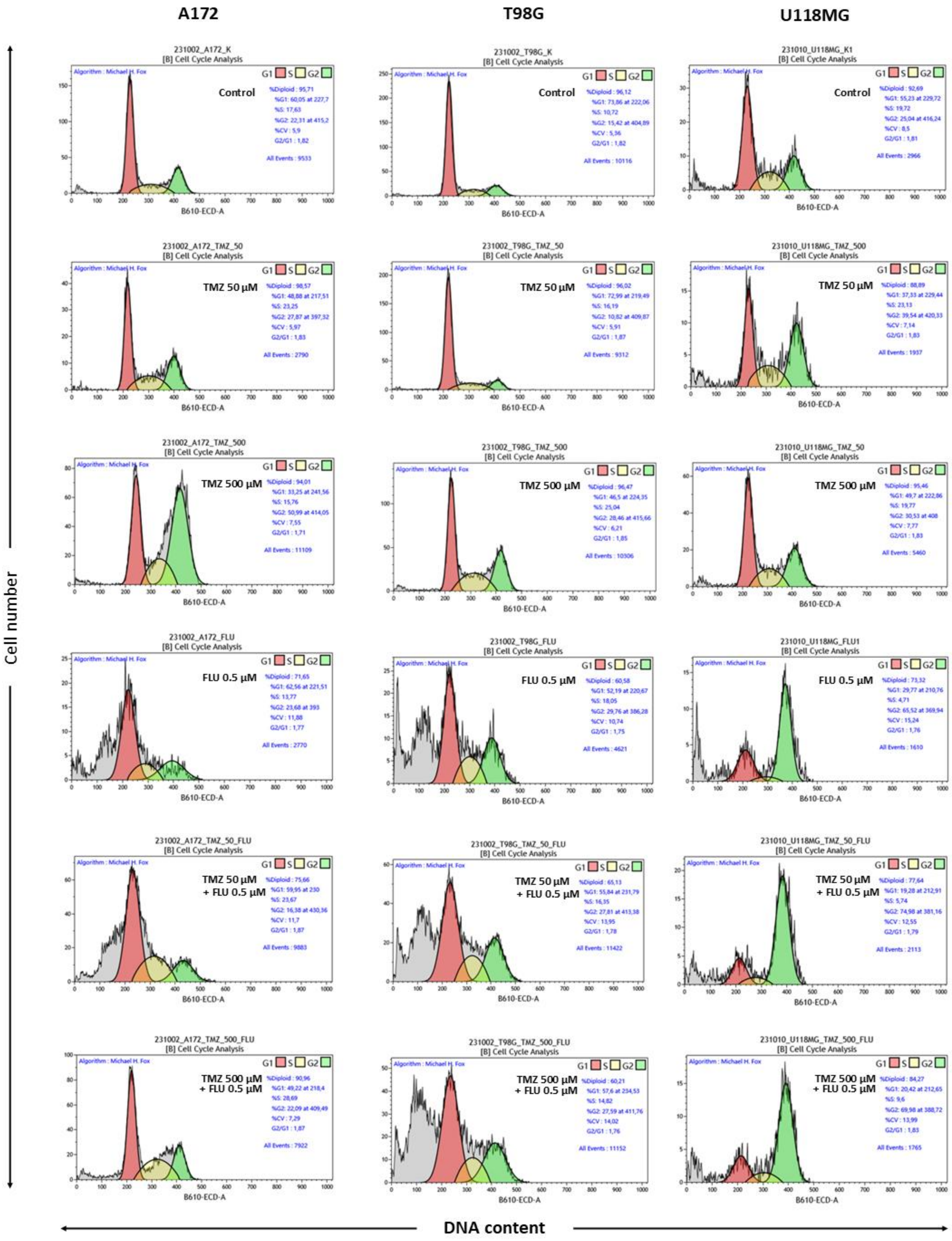

Supplementary Figure S3 Representative PI flow cytometry histograms used for cell-cycle analysis of A172, T98G and U118MG cells following TMZ, FLU and combined treatment.

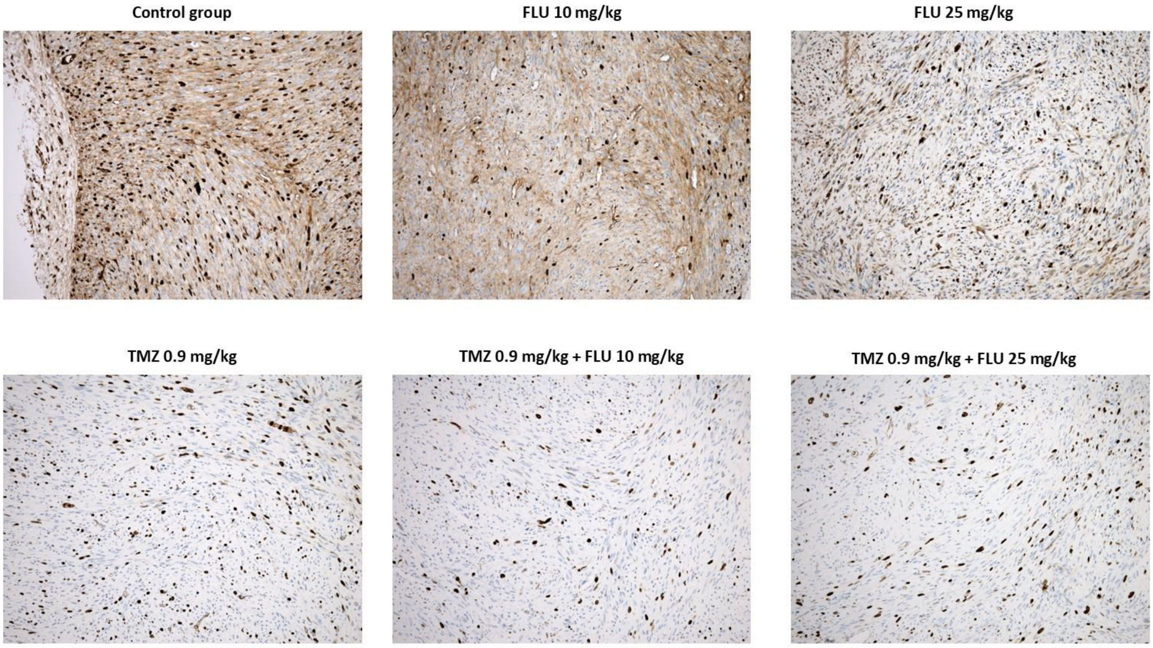

Supplementary Figure S4 Representative images from immunohistochemical analysis of Ki-67 expression in tumors collected from treated athymic nude mice with implanted U118MG glioma cells. 14 days after implantation, the mice were treated p.o. with either FLU 10 mg/kg, FLU 25 mg/kg, TMZ 0.9 mg/kg, TMZ 0.9 mg/kg + FLU 10 mg/kg or TMZ 0.9 mg/kg + FLU 25 mg/kg daily for 14 days. The magnification was 100x.
